# Chronotype, Sleep, and Attention Levels Among Undergraduate Medical Students in South India: A Cross-Sectional Study

**DOI:** 10.7759/cureus.114167

**Published:** 2026-08-08

**Authors:** Sivagami Valliammai, Devasena Srinivasan, Vanishree Shriraam

**Affiliations:** 1 Obstetrics and Gynaecology, SRM Medical College Hospital and Research Centre, Chennai, IND; 2 Internal Medicine, Sri Ramachandra Institute of Higher Education and Research, Chennai, IND; 3 Community Medicine, Sri Ramachandra Institute of Higher Education and Research, Chennai, IND

**Keywords:** academic performance, attention, chronotype, circadian rhythm, cognition, medical students, sleep

## Abstract

Background: Chronotype refers to an individual's preferred timing for sleep and daily activities within a 24-hour cycle. It is influenced by the endogenous circadian rhythm. This study aims to determine chronotype prevalence, sleep, and the association with attention among medical students.

Methods: Undergraduate medical students at a university were asked to fill in two validated questionnaires, including (i) the Morningness-Eveningness Questionnaire for chronotype and (ii) the Mindful Attention Awareness Scale for attention, along with a pre-tested questionnaire for background information and other influencing factors. Data were analyzed using SPSS software version 20.0 (IBM Corp., Armonk, NY). Proportions were calculated, and a test of significance for difference in proportions was calculated using the chi-square test.

Results: A total of 976 students participated in the study. Of them, 67% were females with a mean age of 19.1 (SD ±1.4) years. The most common chronotype was intermediate (655, 67.1%), followed by moderately evening (172, 17.6%) and moderately morning (134,13.7%). Late bedtime, extreme hours of sleep, staying in a hostel, and perceived academic pressure were significantly associated with evening chronotype (p < 0.05). Attention levels were mostly moderate (491, 50.3%) or high (474, 48.6%), with only 11 (1.1%) reporting low attention. High attention was significantly associated with morning chronotype, earlier sleep times, six to eight hours of sleep, day-scholar status, and lower academic pressure (p < 0.05).

Conclusion: Students with a morning chronotype showed a significantly higher proportion of high attention levels. Sleep timing, duration, and perceived academic pressure are important factors affecting both chronotype and attention. Tailoring academic schedules to align with students’ chronotypes could potentially improve attention and thus cognitive performance.

## Introduction

Humans can synchronize their daily activities with environmental signals such as light, temperature, and human interactions through internal rhythms, especially the circadian rhythm, which is regulated by the suprachiasmatic nucleus (SCN) - the body’s master clock [1]. The SCN serves as a critical regulator of sleep and daily activity timing.

Individual differences in preferred timing of sleep and daily activities can be assessed by estimating chronotype as morning, evening, or intermediate [2]. Compared to morning types, evening types typically show sleep patterns delayed by two to three hours [3]. Intermediate types usually rise with the sun, have standard work schedules, flexible sleep timings, and are most active during late morning and early afternoon with the highest productivity. During adolescence and early adulthood, sleep and wake times tend to shift later, influenced by a combination of environmental, psychosocial, and biological factors [2]. These shifts in activity timing may alter circadian alignment, leading to later bedtimes and shorter sleep duration. Lack et al. posit that evening-type persons are more vulnerable to delayed sleep phase disorders [2].

Attention is a core neuropsychological process by which an individual can focus selectively on a stimulus, sustain that focus over time, and shift it at will. Valdez hypothesized that rhythms in cognitive processes maintain a phase relationship with the body’s core temperature rhythm (circadian rhythm) [3].

When irregular sleep patterns disrupt this circadian rhythm, it affects attention, which in turn impacts daily functioning. Thus, sleep quality and quantity are closely linked to students’ learning capacity and academic performance [4,5]. Indeed, numerous studies have explored the association between chronotype and academic performance [6,7]. Among the various student-related determinants of academic engagement, such as learning motivation, interest, self-directedness, mental focus, and emotional regulation, attention emerges as a key factor influencing learning outcomes [8]. However, the study by Valdez is the only one that has specifically investigated the effect of circadian rhythm on each phase of attention [3]. Given this gap, the present study aimed to explore associations among different chronotypes, sleep patterns, and attention in medical students.

## Materials and methods

A cross-sectional study was conducted among undergraduate students at a medical university in South India.

Selection and description of participants

Participants included undergraduate students from a medical university in Chennai, Tamil Nadu. Exclusion criteria were students suffering from chronic medical conditions, such as insomnia, or taking medications that affect sleep. All students enrolled in Bachelor of Medicine and Bachelor of Surgery (MBBS) or Bachelor of Dental Surgery (BDS) programs, from the first to the final year, who were present during their lecture sessions were invited after receiving due permissions from authorities over a period of one month in December 2021.

Sample size calculation

Sample size was calculated using a single proportion estimation formula based on a study by Manjareeka et al. on perceived stress, academic achievement, and chronotype among Indian medical students, wherein the prevalence of morning type was found to be 16.7%. With a relative precision of 15% and a 95% confidence level, the minimum sample size was calculated to be 852 [9].

Data collection

Data were collected using a structured, self-administered questionnaire consisting of three components.

Background Characteristics

The data collected included age, sex, place of stay (hostel (dorms)/non-residents), time to bed, hours of sleep, and susceptibility to social and academic pressure. Susceptibility to academic pressure was assessed by the question “Do you face academic pressure?” A "yes" to this was taken as positive for perceived academic pressure. Susceptibility to social pressure was assessed by two questions: “Do you find it difficult to get along with new people?” and “Have you done things that you do not like in order to avoid isolation from your friends?” An answer of “yes” for both questions indicated susceptibility to social pressure, and a "no" for both or one of them was considered “not susceptible.” This susceptibility was based on the perception of the students.

Chronotype Assessment

The Horne-Östberg Morningness-Eveningness Questionnaire, which consists of 19 items, was used [10]. Based on the scores obtained, students were identified as belonging to definitely morning type (70-86), moderately morning type (59-69), intermediate type (42-58), moderately evening type (31-41), or definitely evening type (16-30) [10].

Attention

The Mindful Attention Awareness Scale (MAAS), a 15-item scale, was used, with each item rated on a six-point Likert scale [11]. The average of the 15 items is computed to get a score between 1 and 6, with higher scores indicating higher attention.

Both tools have been previously validated in a similar population. All tools were administered in English to students who attended the designated lecture session; the investigator provided instructions for each question.

Ethics

Ethical clearance was obtained from the Institutional Ethics Committee (CSP/19/AUG/79/279). Written informed consent was obtained from the participants prior to administration of the questionnaire. The procedures followed were in accordance with the ethical standards of the institutional ethics committee on human experimentation and with the Helsinki Declaration of 1975 (revised 2000).

Statistics

Statistical analysis was done using SPSS software version 20.0 (IBM Corp., Armonk, NY). Questionnaire scores were reported as mean ± standard deviation. Prevalence or proportion was calculated, and a test of proportions was performed using the chi-square (X2) test. P < 0.05 was taken as statistically significant.

## Results

A total of 1050 questionnaires were distributed, of which 976 (92.95%) were returned fully completed. Of these, 324 (33.2%) were completed by males, and 652 (66.8%) were from females. The mean age was 19.11 ± 1.4 years, with a range of 18-24 years. In this study, there were 231 (23.7%) BDS students and 745 (76.3%) medical students.

**Figure 1:**
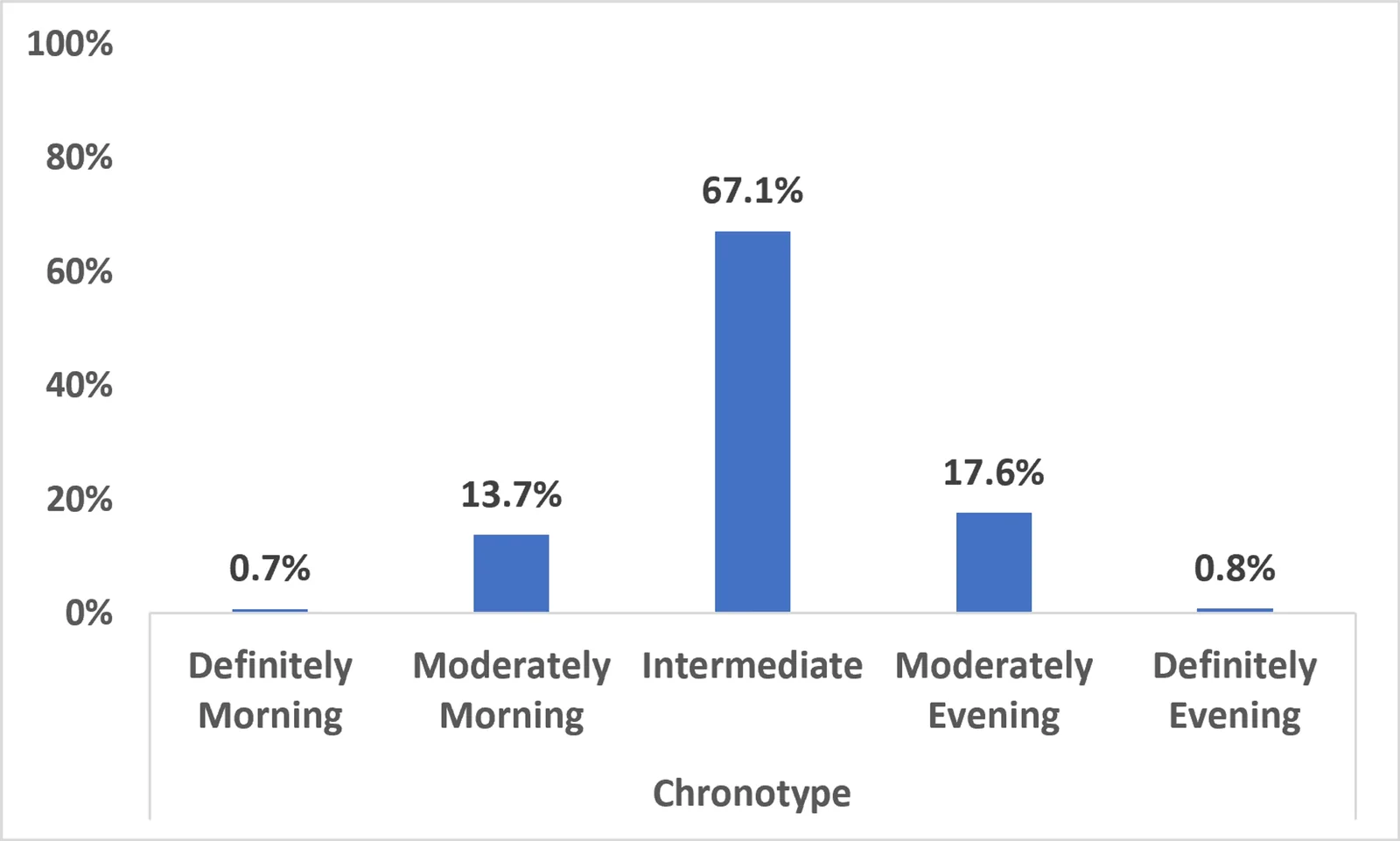
Distribution of chronotype among students in a medical university (n = 976).

The distribution of chronotype among students is shown in Figure 1.

Figure 1 shows the majority of students belonging to intermediate chronotype (655, 67.1%), followed by moderately evening type (172, 17.6%) and moderately morning type (134, 13.7%).

As there were few definitely morning (n = 8) and definitely evening (n = 7) chronotypes, for inferential statistics, the data were grouped into morning, intermediate, and evening. Table 1 shows the association between background variables and the three chronotypes.

**Table 1:** Association of background variables and chronotype (n = 976). * P < 0.05: statistically significant.

Parameters				Morning		Intermediate		Evening		χ² value	P-value
		No.	%	No.	%	No.	%	No.	%		
Sex	Male	324	33.2	46	14.2	225	69.40	53	16.40	1.557	0.459
	Female	652	66.8	95	14.60	430	66	127	19.50		
Age in years	Up to 19	605	62	77	12.70	420	69.30	109	18	4.681	0.096
	20 and above	371	38	64	17.30	235	63.50	71	19.20		
Hours of sleep	<4	14	1.4	1	7.1	7	50	6	42.9	18.478	0.001*
	4-6	297	30.4	31	10.4	194	65.3	72	24.2		
	6-8	559	57.3	95	17	383	68.5	81	14.5		
	8-10	93	9.5	13	14	64	68.8	16	17.2		
	>10	13	1.3	1	7.7	7	53.8	5	38.5		
Time to bed	Before 10 pm	63	6.5	28	44.4	33	52.4	2	3.2	161.696	0.000*
	10 pm to 12 am	580	59.4	106	18.3	408	70.3	66	11.4		
	After 12 am	333	34.1	7	2.1	214	64.3	112	33.6		
Place of stay	Non-residents	451	46.2	81	18	305	67.60	65	14.40	14.588	0.006*
	Residents	525	53.8	60	11.40	350	66.70	11.5	21.90		
Susceptible to social pressure	Yes	373	38.2	47	12.60	254	68	72	19.40	2.219	0.696
	No	603	61.8	94	15.60	401	66.50	108	17.90		
Susceptible to academic pressure	Yes	586	60	63	10.80	394	67.20	129	22	24.009	0.000*
	No	390	40	78	20	261	66.90	51	13.10		

**Figure 2:**
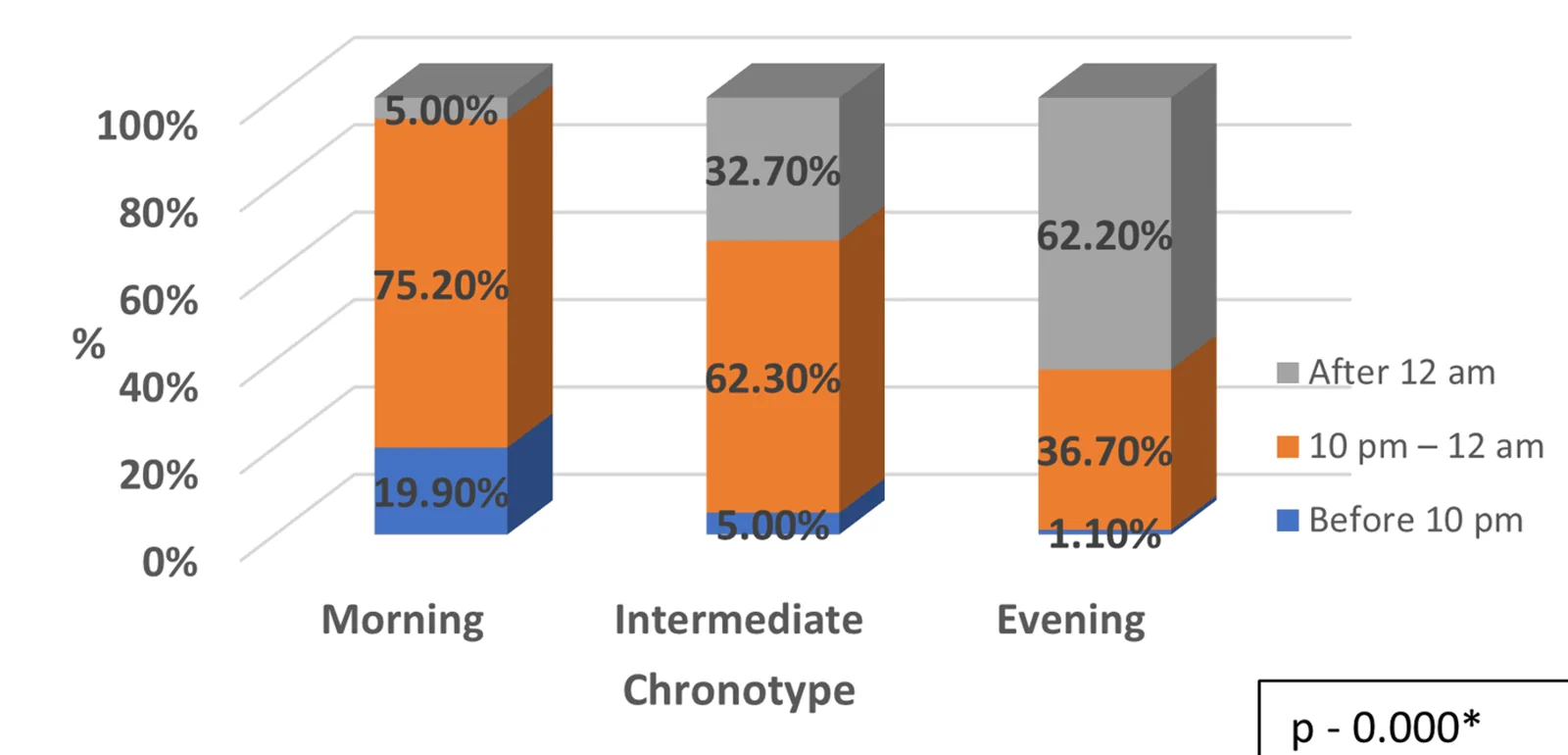
Distribution of the time to bed among students with different chronotypes (n = 976).

The association of age and sex with chronotype was not statistically significant. However, duration of sleep and time to bed were significantly associated with chronotype. Students who slept less than four hours and more than 10 hours were more likely to belong to the evening chronotype (X2 = 18.478; p < 0.001). In addition, those who went to bed after 12 am were mostly evening chronotypes, and those who slept before 10 pm were largely morning types (X2 = 161.696; p < 0.001). A higher proportion of students who stayed in hostels (residents) were of evening chronotype compared to non-residents who stayed with family (X2 = 14.588; p = 0.006). A higher percentage of students who felt susceptible to academic pressure were of evening chronotype (22% vs. 10.8%; X2 = 24.009; p < 0.001). However, a similar difference was not found for social pressure susceptibility and chronotypes (p = 0.696). The distribution of time to bed among students with different chronotypes is shown in Figure 2.

Table 2 presents the association between students’ background characteristics and mean attention scores. Mean attention scores differed significantly across chronotype groups, with morning-type students having the highest mean attention score (4.32 ± 0.83), followed by intermediate (4.02 ± 0.85) and evening chronotypes (3.88 ± 0.82) (p < 0.001). Sleep duration was also significantly associated with attention scores (p < 0.001), with students sleeping six to eight hours demonstrating the highest mean attention score (4.13 ± 0.84), whereas those sleeping more than 10 hours had the lowest mean score (2.37 ± 1.13). Mean attention scores were significantly lower among students reporting susceptibility to social pressure (3.81 ± 0.85 vs. 4.19 ± 0.82; p < 0.001) and academic pressure (3.89 ± 0.84 vs. 4.26 ± 0.82; p < 0.001). The difference in mean attention scores between students was not statistically significant according to sex (p = 0.122), age (p = 0.279), time to bed (p = 0.242), or place of stay (p = 0.053).

**Table 2:** Association between various background factors and attention score.

Variable	Category	Attention score (Mean ± SD)	P-value
Chronotype	Morning	4.32 ± 0.83	<0.001
	Intermediate	4.02 ± 0.85	
	Evening	3.88 ± 0.82	
Sex	Male	3.98 ± 0.86	0.122
	Female	4.07 ± 0.85	
Age (years)	<20	4.02 ± 0.84	0.279
	≥20	4.08 ± 0.87	
Hours of sleep	<4	3.77 ± 1.03	<0.001
	4-6	4.00 ± 0.88	
	6-8	4.13 ± 0.84	
	8-10	3.89 ± 0.81	
	>10	2.37 ± 1.13	
Time to bed	Before 10 pm	4.04 ± 1.04	0.242
	10 pm-12 am	4.10 ± 0.85	
	12-2 am	3.93 ± 0.87	
	After 2 am	3.86 ± 0.90	
Place of stay	Non-resident	4.10 ± 0.82	0.053
	Resident	3.99 ± 0.87	
Susceptibility to social pressure	Yes	3.81 ± 0.85	<0.001
	No	4.19 ± 0.82	
Susceptibility to academic pressure	Yes	3.89 ± 0.84	<0.001
	No	4.26 ± 0.82	

## Discussion

This cross-sectional study assessed the prevalence of chronotypes and sleep patterns, as well as their association with attention scores among 976 undergraduate medical students in South India. The prevalence of intermediate chronotype was the highest (67.1%). This was in accordance with previous studies from similar populations [12-14]. This higher predominance of intermediate followed by evening types in young populations is likely due to developmental shifts in circadian phase, which lead to late sleep onset and wake times [5,15,16].

In contrast to previous studies that have suggested a steady displacement to a late chronotype from ages 11-21, the present study did not find a difference in chronotype prevalence between the younger and older students. In addition, more female students were evening types than males in the current study, though this difference was not statistically significant. This contrasts with the majority of studies, in which males are found to be more evening types in younger years [5,15,17]. However, in some studies, these differences diminished in young adulthood with changing environmental and social influences.

Chronotype’s association with sleep duration and time to bed was statistically significant. The findings of shorter and excessive sleep duration are similar to a sleep study done among students from 26 countries [18]; however, a higher proportion of students reported an average of seven hours of sleep in the current study (57% vs. 47%). Those with extreme sleep duration (<4 hours and >10 hours) as well as late sleepers (after 12 am) had higher rates of evening chronotype, while morning chronotypes had more regular sleep schedules. Multiple studies have reported similar findings among evening chronotype students and earlier bedtimes and earlier rise times among morning types [19,20]. In addition, some have reported much shorter sleep duration during weekdays and longer sleep duration on the weekends among evening chronotypes [2,20-24]. Lack et al. assessed endogenous circadian rhythm among morning and evening chronotype people in a laboratory and found that evening-type individuals typically have an onset of sleep, arising time, and objective sleepiness two to three hours later than morning chronotypes [2].

Place of stay (hostel residence) was significantly associated with evening chronotype, indicating greater autonomy in sleep timing and reduced parental influence. Living with parents usually involves structured bedtime and waking time and some restraint on late-night activities or screen time. However, as students move to dorms, there is a shift toward evening types [25]. Perceived academic pressure, as well as higher exposure to academic stressors, can result in delayed sleep onset and waking time. These factors are not adequately considered in most studies.

In the current study, students’ mean attention score was around 4, which was similar to findings from other studies. Attention was significantly associated with chronotype and hours of sleep. Morning chronotypes and those with adequate sleep duration (six to eight hours) had significantly higher attention scores compared to evening types and those with sleep duration less than four hours and more than 10 hours. A study from China that included 22,000 participants found that a sleep duration of six to seven hours and a bedtime of 10-11 pm are optimal for good attention and academic performance, which is in line with the findings of the current study [26].

Attention scores were higher among students who did not face academic pressure compared to students who faced academic pressure. This finding aligns with a previous study by Lin et al. [27], which found that academic stress is a prevalent form of chronic stress among students and has a notable impact on working memory, particularly within the frontoparietal attention network.

The association between attention and chronotype revealed that morning types had significantly higher attention scores than evening types. This finding is similar to various studies that have explored the association between chronotype and academic performance [6,28,29]. Some of these have found that evening-type students are likelier to fail difficult courses [11,28]. This association supports the synchrony effect, which suggests that cognitive performance peaks when task timing aligns with an individual’s circadian preference. Indeed, multiple studies have shown that morning chronotypes benefit from early-day class schedules [30], while evening types show attention deficits leading to lower academic outcomes and excessive daytime sleepiness [18,26]. If class timings are flexible, those with an evening chronotype will have better alignment with their internal rhythms, higher attention for a longer duration, and better cognitive performance.

The strengths of the present study include its large sample size and use of validated tools to assess chronotype and attention. However, the study design was cross-sectional, so causal explanations were not possible. Other limitations include lack of objective sleep measures, use of limited questions to assess academic/social pressure, and absence of multivariable analysis to control for confounders. The use of self-reported questionnaires may have led to reporting bias, social desirability bias, etc. Finally, this study focused on a single institution, limiting its generalizability. Future research on this topic should implement objective sleep measures and longitudinal designs to better understand the temporal relationship between chronotype, sleep, attention, and cognitive performance.

## Conclusions

This study illustrates the association between chronotype, sleep habits, and attention levels. The majority of evening chronotype students went to bed very late and had extreme sleep duration. They also had higher perceived academic pressure compared to morning types. Morning types had a higher proportion of high attention levels than evening-type students; these differences were statistically significant.
